# MMP-9 and CXCL8/IL-8 Are Potential Therapeutic Targets in Epidermolysis Bullosa Simplex

**DOI:** 10.1371/journal.pone.0070123

**Published:** 2013-07-19

**Authors:** Thomas Lettner, Roland Lang, Alfred Klausegger, Stefan Hainzl, Johann W. Bauer, Verena Wally

**Affiliations:** 1 Division of Experimental Dermatology and EB House Austria, Salzburg, Austria; 2 Department of Dermatology, Paracelsus Medical University, Salzburg, Austria; University of Vienna, Max F. Perutz Laboratories, Austria

## Abstract

Epidermolysis bullosa refers to a group of genodermatoses that affects the integrity of epithelial layers, phenotypically resulting in severe skin blistering. Dowling-Meara, the major subtype of epidermolysis bullosa simplex, is inherited in an autosomal dominant manner and can be caused by mutations in either the keratin-5 (*K5*) or the keratin-14 (*K14*) gene. Currently, no therapeutic approach is known, and the main objective of this study was to identify novel therapeutic targets. We used microarray analysis, semi-quantitative real-time PCR, western blot and ELISA to identify differentially regulated genes in two *K14* mutant cell lines carrying the mutations K14 R125P and K14 R125H, respectively. We found kallikrein-related peptidases and matrix metalloproteinases to be upregulated. We also found elevated expression of chemokines, and we observed deregulation of the Cdc42 pathway as well as aberrant expression of cytokeratins and junction proteins. We further demonstrated, that expression of these genes is dependent on interleukin-1 β signaling. To evaluate these data *in vivo* we analysed the blister fluids of epidermolysis bullosa simplex patients vs. healthy controls and identified matrix metalloproteinase-9 and the chemokine CXCL8/IL-8 as potential therapeutic targets.

## Introduction

Epidermolysis bullosa (EB) is a genetically heterogeneous disease affecting the skin and mucous membranes. EB is characterized by the formation of blisters and erosions after minor traumatization, thereby significantly compromising life quality. EB is divided into four major groups: the simplex type (EBS), the dystrophic type (DEB), the junctional type (JEB) and Kindler syndrome. The genes underlying the different subtypes of EB have major functions in mechanical stabilization of the basement membrane zone. Depending on the gene which is affected, EB can be either a relatively mild disease or a life-threatening disease due to secondary complications like squamous cell carcinomas in dystrophic EB, in which the collagen VII (*COL7A1*) gene is mutated [1].

In the EBS subtype, mutations in the keratin-5 (*K5*), keratin-14 (*K14*) and plectin (*PLEC*) genes are causative, with many being inherited as autosomal dominants and therefore presenting a challenge to gene therapy. EBS type Dowling-Meara (EBS-DM) is caused by such dominant mutations in the *K5* and *K14* genes and belongs to the more severe subtypes within the EBS group [2].

The type-II keratin K5 and the type-I keratin K14 are the major components of the intermediate filament (IF) network in basal cells of epithelia, forming heterodimers that are bundled as tonofilaments. These IFs are attached to desmosomes and hemidesmosomes and provide mechanical stability not only within a single cell but, also between neighboring cells and to the basement membrane [3]. Due to the dominant nature of K5 and K14 mutations in EBS-DM, misfolded proteins can be integrated into the IFs, rendering them sensitive to mechanical stress. Upon trauma, these filaments disrupt and the keratinocytes lyse, leading to intra-epidermal blistering [4].

Yet, the function of IFs is considered to be more than just to provide mechanical stability to basal keratinocytes. It was shown that, upon mechanical stress, major MAPK pathways like ERK are activated in K14 mutant cell lines and change the apoptotic machinery within these cells [5]. Another form of stress response was shown in K14 mutant cell lines and in a K5^−/−^ mouse model for EBS. In the latter, the inflammatory cytokines IL-6 and IL-1β were found to be upregulated in K5^−/−^ mouse skin and it was hypothesized that keratin mutations contribute to EBS by inducing an inflammatory phenotype that mediates a stress response [6].

An important role of IL-1β in the skin is to activate keratinocytes in many pathological conditions and upon wounding. In basal keratinocytes, IL-1β is present in the cytoplasm in a precursor form. After injury, IL-1β is processed and released and activates signal transduction pathways in surrounding cells in both autocrine and paracrine fashion. In keratinocytes, IL-1β alters gene expression and causes cells to become proliferative and migratory [7].

Based on the fact that many stress pathways are activated in K14 mutant cells, we hypothesized that these pathways contribute to the blistering phenotype of EBS-DM patients to a greater extent than is usually supposed. In the present study, we investigated the gene expression profiles of two EBS-DM cell lines and compared them to that of a wild-type cell line. In a hypothesis-driven as well as hypothesis-generating approach, we identified a plethora of regulated genes in these cell lines. We investigated the relevance of these genes *in vivo*, and our data illuminate potential therapeutic targets that may provide a basis for future medical treatments.

## Materials and Methods

### Ethics Statement

In the course of this study we used a punch biopsy of a five-year-old patient to generate the immortalized cell line EBDM-1 (see below). The biopsy was taken for diagnostic reasons, and the parents gave written informed consent to use the remaining material for scientific research. The Salzburg ethics committee confirmed, that in this case no ethics approval is necessary, and that the procedure is in accordance with the Krankenanstalten- und Kuranstaltengesetz, §8c (Austrian Federal Hospital Act, section 8c), and with the Salzburger Krankenanstaltengesetz 2000, §30 (Salzburg Hospital Act 2000, section 30). No institutions or hospitals outside of Austria were associated with research on primary patient material. Samples of totalRNA of established cell lines were processed outside of Austria for microarray analysis by a commercial service (see below). For the microarray analysis, the cell lines were anonymized and could not be linked to certain individuals. Therefore, all ethical issues fall under Austrian legislation.

### Cell Lines and Cell Culture

All cell lines used in this study were immortalized in the same way with HPV16 E6/E7. NEB-1 and KEB-7 cell lines were generously provided by the laboratory of E.B. Lane, College of Life Sciences, University of Dundee. KEB-7 cells are keratinocytes derived from a Dowling-Meara patient carrying the K14 R125P mutation (coding sequence G375C) and NEB-1 cells are wild-type keratinocytes derived from a healthy relative of this patient [8]. EBDM-1 cells were obtained from a skin biopsy of a five-year-old Dowling-Meara patient heterozygous for a K14 R125H mutation (coding sequence G375A). The skin biopsy for EBDM-1 cells was performed at the Dermatology Department of Paracelsus Medical University Salzburg (see ethics statement). The primary keratinocytes were isolated by incubating the biopsy in trypsin-EDTA for 30 minutes and transferring the epidermis onto a feeder layer in Epilife® medium (Invitrogen). Immortalized keratinocyte cell lines were cultured in RM medium (DMEM plus 25% Ham’s F12 medium, 10% heat-inactivated FCS, 1% Pen/Strep and additional growth factors: adenine 1.8×10^−4^ M, hydrocortisone 0.4 µg/ml, transferrin 5 µg/ml, lyothyronine 2×10^−11^ M, insulin 5 µg/ml and EGF 10 ng/ml). All cell lines were incubated at 37°C, 5% CO_2_ in a humidified atmosphere. All experiments were performed within comparable passages and at 70% confluence. For interleukin-1β experiments, human IL-1β/IL-IF2 antibody polyclonal goat IgG (R&D Systems, # AB-201-NA) was added to the culture medium at a final concentration of 2 µg/ml of medium.

### Microarray Analysis

According to MIAME guidelines [9], the microarray was performed as follows: NEB-1 and KEB-7 cells of passage 20 and EBDM-1 cells of passage 13 were harvested at 70% confluence and total RNA was extracted from cell lysates using an RNeasy Mini Kit (Quiagen, # 74104) according to the manufacturer’s protocol. Sample processing and data analysis was performed by an Affymetrix Service Provider and Core Facility, “KFB-Center of Excellence for Fluorescent Bioanalytics” in Regensburg, Josef-Engert-Straße 9, D-93053 Germany. At KFB, an Ambion® WT Expression Kit was used to generate sense-strand cDNA from total RNA of NEB-1, KEB-7 and EBDM-1 samples according to the manufacturer’s protocol. The sense-strand cDNA was then fragmented, labelled and hybridized using the Affymetrix GeneChip® WT Terminal Labeling and Hybridization Kit according to the manufacturers protocol. For data analysis, the Affymetrix Expression Console Software was used, and the Robust Multi-chip Analysis (RMA) algorithm was applied with default settings. The microarray dataset was submitted to ArrayExpress (http://www.ebi.ac.uk/arrayexpress/) as two separate experiments (accession number KEB-7 vs. NEB-1: E-MTAB-1640; accession number EBDM-1 vs. NEB-1: E-MTAB-1641). The bioinformatic online tool DAVID (http://david.abcc.ncifcrf.gov/) was used for further analysis of the microarray dataset [10]. In order to obtain statistically significant expression data, the expression of regulated target genes, identified in the microarray, was confirmed with SQRT-PCR. Targets were chosen for further investigation with SQRT-PCR even when they were found to be regulated in only one of the two EBS-DM cell lines. The microarray data, relevant to the present study, are given in detail in the results section, including the Sig log ratio, the fold expression and the mRNA accession number (Ref Seq) for each identified gene. The Sig log ratio is the difference in the log2 signal of a probe compared between two arrays (e.g. gene X in KEB-7 vs. NEB-1). A Sig log ratio of +1 is equivalent to two-fold upregulation. A Sig log ratio of −1 is equivalent to two-fold downregulation. A Sig log ratio of 0 indicates no change.

### Semi-quantitative Real-time PCR (SQRT-PCR)

Cells were harvested at 70% confluence and total RNA was extracted from cell lysates using an RNeasy Mini Kit (Quiagen, # 74104) according to the manufacturer’s protocol. DNase1 digestion (DNase1, Amplification Grade, Sigma-Aldrich, # AMPD1-1KT) and cDNA synthesis (iScript™ cDNA Synthesis Kit, *BIO-RAD*, # 170-8891) were also performed following the manufacturers’ protocols. Primers were designed to bind over exon-exon junctions to exclude binding to intronic sequences and to amplify an equal product length of 150 bp for all target genes. *GAPDH* was used as a reference gene. SQRT-PCR was performed using iQ SYBR Green Supermix (*BIO-RAD*, # 170-8882) in a *BIO-RAD* CFX96™ Real-Time System, C1000™ Thermal Cycler. A three-step protocol was used, and the 2^−ΔΔCt^ method was applied for quantification of gene expression [11]. All SQRT-PCR results are given in fold expression. Primer sequences were as follows: ARHGEF9-fw-AAGACCACAGTGACTACAGG, ARHGEF9-rv-TCCTCGCCCTCCCAGTCT, CXCL1-fw-CGCGCAGCAGGAGCGTC, CXCL1-rv-ATTCTTGAGTGTGGCTATGAC, CXCL11-fw-AGTTGTTCAAGGCTTCCCCA, CXCL11-rv-TTCAGGGTAATAATCACTTCTAT, CXCL14-fw-TGTGGACGGGTCCAAATGC, CXCL14-rv-CCTGACCTCGGTACCTGG, DSC1-fw-TAGAGAAAAATGTGATACTTACC, DSC1-rv-GTAACATAAGAAGTTTCTGTGAA, DSC2-fw-CAGAGAGTTAATTGACAAGTAC, DSC2-rv-GTCACATAAGAAGTACGAGTAA, DSC3-fw-ACAGAGAGGTTGTAGACAAGT, DSC3-rv-CTTCATAAGCATTTTGTCTGAAA, DSG1-fw-CAGAGAGCAATACGGCCAG, DSG1-rv-ATGGTATATGAAGACTGTTCCA, DSG3-fw-ACCCTCAATGCTACCTCGG, DSG3-rv-TGTTGTCACACTGACAGACTT, DSG4-fw-CAACAGATGTCAGATATATCATA, DSG4-rv-CTATAGCCAGGATCTCTGCT, GAPDH-fw-GCCAACGTGTCAGTGGTGGA, GAPDH-rv-CACCACCCTGTTGCTGTAGCC, GJA1-fw-GACATGCACTTGAAGCAGATT, GJA1-rv-CTGGATCAGCAAGAAGGCC, GJB2-fw-CTATGGGCCCTGCAGCTG, GJB2-rv-CCTTCTGGGTTTTGATCTCC, GJB6-fw-GGGCCCTCCAGCTGATCT, GJB6-rv-GAACCTTCTGCTTTTTAATGTC, IL8-fw-TGTGAAGGTGCAGTTTTGCC, IL8-rv-AAGCTTTACAATAATTTCTGTGTT, KLK5-fw-AGTCAGAAAAGGTGCGAGGA, KLK5-rv-TGAACTTGCAGAGGTTCGTG, KLK7-fw-TCAAGGCCTCGAAGTCATTC, KLK7-rv-GGTCAGAGGGAAAGGTCACA, KRT5-fw-TCTCGCCAGTCAAGTGTGTC, KRT5-rv-ATAGCCACCCACTCCACAAG, KRT6b-fw-ACCAGACAAAGTACGAGGAG, KRT6b-rv-TGTAGGTTGGCACACTGCTT, KRT8-fw-TCATCAAGAAGGATGTGGATG, KRT8-rv-ACCACAGATGTGTCCGAGAT, KRT14-fw-TTCTGAACGAGATGCGTGAC, KRT14-rv-GCAGCTCAATCTCCAGGTTC, KRT15-fw-GAGAACTCACTGGCCGAGAC, KRT15-rv-CTGAAGAGGCTTCCCTGATG, KRT16-fw-GAGGAACAAGATCATTGCGG, KRT16-rv-CGCAGGCCATTGACGTCGG, KRT17-fw-TTCTTCAGCAAGACAGAGGAA, KRT17-rv-AGGGATGCTTTCATGCTGAG, KRT18-fw-CGTTTCTGGGGGCATGAGCTTCACC, KRT18-rv-AAGAGGCCAGGCGGTCGTTC, MMP1-fw-TCAGGGGAGATCATCGGGA, MMP1-rv-GATGTAAGTTGTACTCTCTGAA, MMP7-fw-GAGTGCCAGATGTTGCAGAA, MMP7-rv-GCCAATCATGATGTCAGCAG, MMP9-fw-GGTGTCGCGGAGCACGG, MMP9-rv-GAGTTGGAACCACGACGCC, MMP13-fw-TTGAGCTGGACTCATTGTCG, MMP13-rv-GGAGCCTCTCAGTCATGGAG, MMP19-fw-GGCTCTCTATGGCAAGAAGA, MMP19-rv-CCACGGGGCCCCAGCAT, WIPF1-fw-GCCGATCAGGCCCCCTC, WIPF1-rv-TGGGCCTATCAGGAGGAAG.

### Isolation of Total Protein from Cell Cultures

Cells were grown to 70% confluence and then washed twice with PBS. The cells were covered with lysis buffer (0.5 M Tris-HCl -pH 6.8, 20% glycine, 10% SDS, 5% ß-mercaptoethanol and Roche complete protease inhibitor), scraped off with a rubber policeman and transferred into microcentrifuge tubes. The cells were lysed by pipetting three times through a 22G syringe and then incubated at 95°C for five minutes. The cell lysates were stored at –20°C.

### Isolation of Proteins from Cell Culture Supernatant

For western blot analysis of kallikrein-related peptidases, cells were incubated for 48 h in Epilife® medium without FCS. The supernatant was collected and filtered through a cell strainer (BD Falcon™) to remove dead cells and cell debris. Complete mini protease inhibitor (Roche) was added to the supernatant and the solution was concentrated by centrifugation through a centrifugal filter (Amicon® Ultra, Millipore™) for 20 minutes at 4°C and 6800 g. The concentrate was mixed with 4× sample buffer and subject to SDS-PAGE and western blotting as described below.

### Collection of Blister Fluid

Patients of various EBS subtypes visited the Dermatology Department of Paracelsus Medical University and the EB house Austria for routine check up and wound care. In the course of wound care, blister fluid was collected with a syringe. Roche complete protease inhibitor was added to the samples and aliquots of 1/10 and 1/20 dilutions were stored at –80°C. Control samples of three otherwise healthy volunteers were treated in the same way. Table 1 summarizes the data of patients and healthy controls.

**Table 1:** Summarized patient data.

	Patient	Affected gene and mutation	P*	Age**	Blister location
**1**	MB	Healthy control	–	76	Left inguinal
**2**	MB	Healthy control	–	42	Left and right heel
**3**	BB	Healthy control	–	34	Left foot
**4**	EBS	K14, CD	mi	72	Pooled***
**5**	EBS	K14, CD	mi	13	Pooled***
**6**	EBS-WC	K14, CD	mi	17	Right foot
**7**	EBS-WC	K14, CD	mi	6	Left foot
**8**	EBS-K	K14, Y248X, 744delC/insAG, ex3	mi	11	Left shoulder
**9**	EBS-DM	K14, CD	me	1	Right thigh
**10**	EBS-DM	K14, E411K, 1231G>A, ex6	se	5	Left calf
**11**	EBS-MD	PLEC1, 954–956 dupGCT, ex9;	me	13	Left hand
		PLEC1, Q1408X, 4222C>T, ex31			

* P, phenotype: mi, mild; me, medium; se, severe.

** Age at time of sample collection.

*** Two or more blisters collected from different locations and pooled. Abbr.: BB, burn blister; CD, clinical diagnosis; del, deletion; dup, duplication; EBS-DM, EBS Dowling-Meara; EBS-K, EBS Koebner (EBS generalized other); EBS-MD, EBS with muscular dystrophy; EBS-WC, EBS Weber-Cockayne (EBS localized); ex, exon; ins, insertion; MB, mechanical blister.

### Western Blot

Protein levels were determined by SDS-PAGE and western blot analysis on NuPAGE® 10% Bis-Tris Gels (Invitrogen) using 20 µl cell lysate or concentrated cell culture supernatant of each determined cell line. Annexin-I was used as a loading control for cell lysates. As a negative control for the western blot, one randomly chosen extra sample was applied; all gels included lanes with 4 µl of size marker (*BIO-RAD* Precision Plus Protein™ WesternC™ Standards). The electrophoresis run was performed at 100 Volts for about 2 h in 1× NuPAGE® buffer (Invitrogen). The size-separated proteins on the polyacrylamide gel were then electrophoretically transferred to a nitrocellulose membrane (Amersham™ Hybond™-ECL, RPN78D). Blotting was done at 4°C at 250 mA for 1 h in transfer buffer (Tris-base 25 mM, glycine 192 mM, methanol 20%). The membrane was then incubated in blocking solution (5% low-fat dry milk powder diluted in 1× TBS containing 0.2% Tween 20) for 1 h at room temperature to saturate unspecific antibody-binding sites. The blocking reagent was then discarded and the primary antibody was applied (Cytokeratin-14, mouse monoclonal IgG, Santa Cruz Biotechnology, diluted 1/1000 in blocking reagent; Cytokeratin-15, rabbit monoclonal IgG, abcam, diluted 1/10,000 in blocking reagent; Cytokeratin-16, goat polyclonal IgG, Santa Cruz Biotechnology, diluted 1/500 in blocking reagent; phospho-ERM, rabbit polyclonal, Cell Signalling, diluted 1/500 in blocking reagent; KLK5 antibody, goat polyclonal IgG, abcam, diluted 1/100 in blocking reagent; KLK7 antibody, rabbit polyclonal IgG, abcam, diluted 1/100 in blocking reagent; Annexin-I, mouse monoclonal IgG, Santa Cruz Biotechnology, diluted 1/1000 in blocking reagent). Primary antibodies were incubated at 4°C over-night. No primary antibody was applied to the negative control. The next day, the primary antibody solution was washed away with blocking solution three times for ten minutes at room temperature. The secondary antibodies (Goat anti-mouse IgG:HRP, Serotec, 1/1000 in blocking solution; Goat anti-rabbit IgG:HRP, abcam, 1/1000 in blocking solution; Rabbit anti-goat IgG:HRP, Dako, 1/1000 in blocking solution) were then applied to the membrane containing the samples as well as to the negative control. *BIO-RAD* Precision Protein™ StrepTactin-HRP conjugate (1/5000 in blocking solution) was applied to the size marker. The secondary antibodies were incubated for 1 h at room temperature and the membrane was then washed three times for ten minutes with TBS containing 0.2% Tween-20. HRP staining solution was prepared 1∶1 (*BIO-RAD* Immun-Star™ WesternC™ Kit) and applied to the membrane as well as to the negative control and to the size marker. The membrane was placed between two layers of transparent foil and analyzed on a *BIO-RAD* Molecular Imager® ChemiDoc™ XRS system using Quantity one 4.6.5 software.

### Immunoprecipitation of Active (GTP-bound) Cdc42

The evening before the experiment 9×10^5^ cells/well of every cell line were seeded into 6-well plates and incubated in RM medium over night at 37°C, 5% CO_2_ in a humidified atmosphere. All of the following steps were performed on ice or at 4°C. The next day, the medium was discarded and the cells were washed once with ice-cold PBS. 550 µl lysis buffer (25 mM Tris-base, 140 mM NaCl, 1 mM EDTA, 0.5% NP-40 and Roche complete protease inhibitor) were applied per well and the cells were scraped off using a rubber policeman. The 6-well plates were kept on ice during the procedure. The lysates were transferred to 1.5-ml reaction tubes and incubated for 45 minutes at 4°C with constant rotation. The tubes were centrifuged at 15,000 g for 10 minutes at 4°C. The supernatant, containing the cleared lysate, was transferred into a new reaction tube and the pellet discarded. 50 µl of the cleared lysate were saved for determination of the loading control in SDS-PAGE and western blot. 1 µg of the antibody (Anti-active-Cdc42 antibody, mouse monoclonal IgG, NEWEAST Biosciences), was added to the lysate. The antibody/lysate solution was then incubated for 2 h at 4°C with constant rotation. After 2 h, 30 µl of Protein G Sepharose™ 4 Fast Flow (GE Healthcare) were added to the solution and the incubation was continued for 2 h at 4°C with constant rotation. The lysates were then centrifuged at 4°C for 5 minutes at 250 g. The supernatant was discarded and the pellet was washed twice with lysis buffer (without protease inhibitor) then twice with wash buffer (100 mM Tris-base, 0.5 M LiCl) and once with PBS (all solutions ice-cold). Between the washing steps the lysates were centrifuged at 4°C for 5 minutes at 250 g. The pellet was resuspended by flicking the tube gently and 50 µl of 2× sample buffer were added; the pellet was again resuspended by flicking and then boiled for 5 minutes at 95°C. After boiling, the samples were centrifuged for 3 minutes at 11,000 g and 20 µl of each sample supernatant were loaded onto a NuPAGE® 10% Bis-Tris Gel (Invitrogen). SDS-PAGE and western blot were performed as described above with primary antibody (Anti-Cdc42 antibody, rabbit polyclonal, Cell Signaling, diluted 1∶500 in blocking reagent) and Annexin-I loading control (Annexin-I, mouse monoclonal IgG, Santa Cruz Biotechnology, diluted 1/1000 in blocking reagent). Secondary antibodies: Goat anti-mouse IgG:HRP, Serotec, 1/1000 in blocking reagent; Goat anti-rabbit IgG:HRP, abcam, 1/1000 in blocking reagent.

### Determination of Relative Protein Amounts after Western Blotting

In most cases the differences in protein expression, analyzed by western blotting, were visible to the naked eye. Nevertheless, *BIO-RAD* Image Lab 3.0.1 software was used to determine the relative protein amounts of the different cell lines by computationally normalizing the target bands to the loading control bands in all samples and then calculating the relative differences between the target bands of the determined cell lines.

### Enzyme-linked Immunosorbent Assay (ELISA)

Protein levels of KLK5, MMP7, MMP9, CXCL1, CXCL8/IL-8, CXCL11 and CXCL14 were determined in 48-h-conditioned cell culture supernatant and in patients blister fluids by using Quantikine® ELISA (Human KLK5, # DKK500, R&D Systems; Human MMP7, # DMP700, R&D Systems; Human MMP9, # DMP900, R&D Systems; Human CXCL1/GROα, # DGR00, R&D Systems; Human CXCL8/IL-8, # D8000C, R&D Systems; Human CXCL11/I-TAC, # DCX110, Human CXCL14/BRAK, # DY866, R&D Systems) following the manufacturer’s protocol. 50 µl of pure (undiluted) cell culture supernatant were used without addition of protease inhibitors. 50 µl, 100 µl or 200 µl of 1/10 or 1/20 dilutions of blister fluid were used according to the Quantikine® ELISA protocol. Concentrations were measured in ng/ml or pg/ml. Results for patient cell lines are given as fold expression compared to NEB-1 wild-type cells. Results for patients blister fluids are given as concentrations.

### Statistical Analysis

For SQRT-PCR and Quantikine® ELISA experiments, a Student`s *t*-test was performed to determine statistical significance with parameters “two-tailed” and “unpaired”. The number of repeats (n) and p-values (p) are given in detail in every figure legend.

## Results

### Microarray Analysis Revealed Differentially Expressed Genes in the EBS-DM Cell Lines KEB-7 and EBDM-1

Our group recently demonstrated, that IL-1β is a critical determinant of the phenotype of the EBS-DM patient cell lines KEB-7 and EBDM-1 by way of activating the JNK stress pathway [12]. We also showed in the same study that these cells exhibit an invasive phenotype in Matrigel™ invasion chambers [12].

Based on that information, we expected to observe altered gene expression profiles for KEB-7 and EBDM-1 cells compared to NEB-1 wild-type keratinocytes. To identify differentially regulated genes, we performed a whole-genome microarray analysis and compared the transcriptome levels of KEB-7 and EBDM-1 cells to NEB-1 cells. The DAVID bioinformatics tool (http://david.abcc.ncifcrf.gov/) was used to identify regulated genes that are relevant to the disease pathomechanism, especially regarding blister formation and invasiveness, and the genes were grouped into functional classes. Six major functional classes were defined: 1) kallikrein-related peptidases, 2) matrix metalloproteinases, 3) factors related to actin cytoskeleton dynamics, 4) cytokeratins, 5) junction proteins, and 6) chemokines. Genes were chosen as targets for further analysis even if they were regulated in only one of the two EBS-DM cell lines. We used SQRT-PCR to confirm the microarray data at the gene expression level as well as western blot and ELISA to confirm expression at the protein level. In the following sections, we present the microarray data and describe and discuss the possible roles of the identified genes in inducing a blistering phenotype.

### Kallikrein-related Peptidases were Upregulated in EBS-DM Cell Lines but not in Patients Blister Fluids

**Figure 1:**
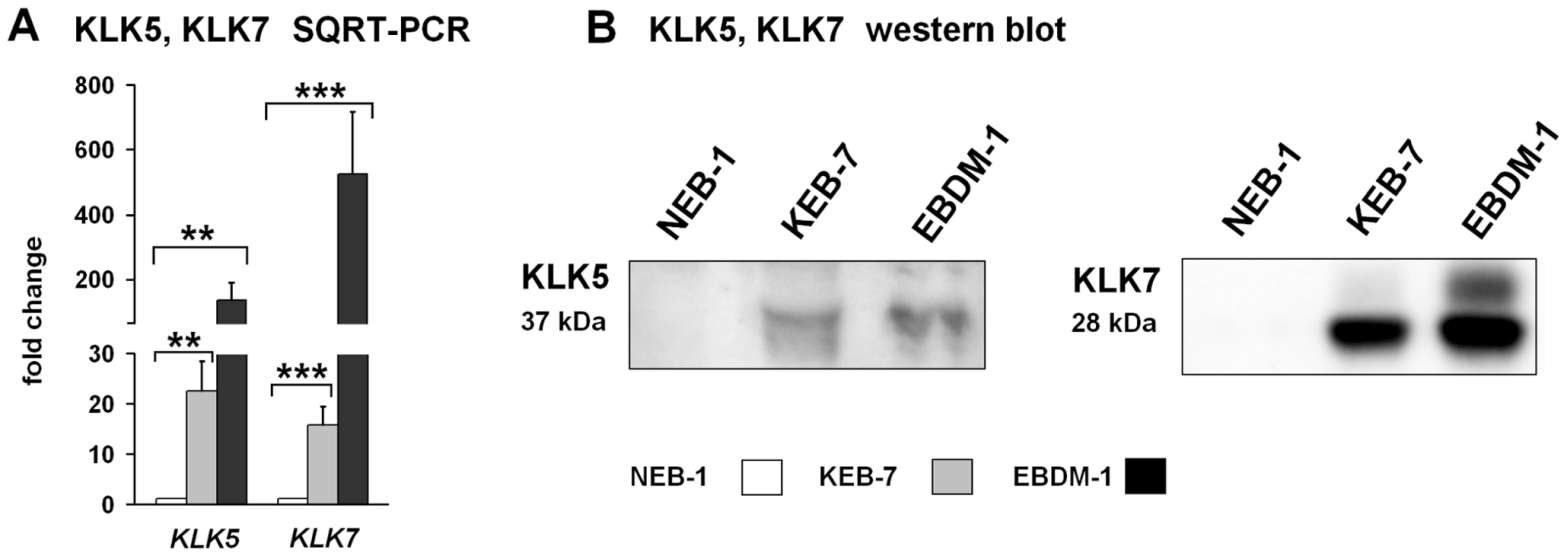
Expression of KLK5 and KLK7. **A.** SQRT-PCR shows increased KLK5 and KLK7 mRNA expression in EBS-DM cell lines (n = 4 to n = 5). **B.** Increased expression of KLK5 and KLK7 protein in EBS-DM cell lines determined in 48-h-conditioned cell culture supernatant by western blot. Student’s *t*-test was performed with p values: ** ≤0.01, *** ≤0.005.

Among the 15 known human kallikrein-related peptidases (KLKs), KLKs 5, 6, 7, 8, 10, 11, 13 and 14 were found by microarray analysis to be significantly upregulated. *KLK5* and *KLK7* showed the highest increase in gene expression in both of the investigated EBS-DM cell lines (Table 2). Both KLK5 and KLK7 are known to contribute to skin desquamation in the stratum corneum by degrading desmosomal proteins [13]. The increased expression of *KLK5* and *KLK7* was confirmed by SQRT-PCR for both KEB-7 and EBDM-1 cells (Fig. 1A). Western blot analysis showed elevated amounts of KLK5 and KLK7 protein in the cell culture supernatant when compared to NEB-1 cells (Fig. 1B). To evaluate the *in vivo* situation, we analysed the blister fluids of EBS patients vs. healthy controls. KLK5 and KLK7 protein levels showed no significant differences between patients and controls, as analysed by ELISA and western blot, respectively (data not shown).

**Table 2:** Microarray analysis of kallikrein-related peptidases.

		KEB7 vs. NEB1		EBDM1 vs. NEB1	
Ref Seq	Gene	Sig logratio	Foldchange	Sig logratio	Foldchange
NM_002257	KLK1	0.49	1.40	−0.15	−1.11
NM_001002231	KLK2	−0.04	−1.03	0.08	1.06
NM_001030047	KLK3	−0.11	−1.08	−0.14	−1.10
NM_004917	KLK4	0.10	1.07	−0.18	−1.13
NM_012427	KLK5	4.83	28.4	2.70	6.48
NM_002774	KLK6	0.37	1.30	2.55	5.86
NM_139277	KLK7	4.19	18.2	2.85	7.23
NM_144505	KLK8	1.58	2.98	1.34	2.53
NM_012315	KLK9	0.71	1.64	0.48	1.39
NM_002776	KLK10	3.60	12.1	0.37	1.29
NM_144947	KLK11	2.41	5.31	0.61	1.52
NM_145894	KLK12	0.08	1.05	0.03	1.02
NM_015596	KLK13	2.26	4.78	0.16	1.12
NM_022046	KLK14	1.49	2.82	0.57	1.49
NM_017509	KLK15	0.11	1.08	0.12	1.08

### Expression of Matrix Metalloproteinase-9 was Increased in EBS-DM Cell Lines and Patients Blister Fluids

There are 23 human matrix metalloproteinases (MMPs) that are either secreted from the cell or membrane-bound. They contribute to a variety of functions, including extracellular matrix (ECM) degradation, tumor invasion, tissue remodeling and embryogenesis [14].

**Figure 2:**
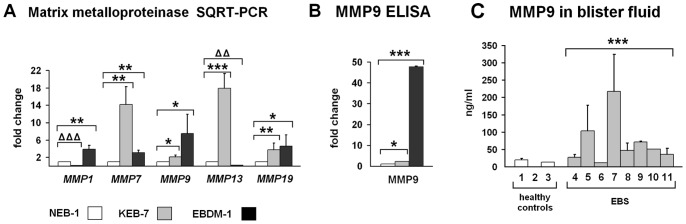
Expression of matrix metalloproteinases. **A.** SQRT-PCR shows increased matrix metalloproteinase mRNA expression in EBS-DM cell lines (n = 4). **B.** MMP-9 ELISA of 48-h-conditioned cell culture supernatant shows 2-fold upregulation of MMP-9 in KEB-7 and 46-fold upregulation in EBDM-1 at the protein level (n = 4). **C.** MMP-9 levels were highly increased in EBS patients blister fluids compared to healthy controls (n = 3 to n = 4). The numbers correlate with Table 1. Student`s *t*-test was performed with p values: * ≤0.05, ** ≤0.01, *** ≤0.005, ΔΔ≤0.0005, ΔΔΔ≤0.0001. (In 2C, the Student’s *t*-test compared the entire patient group to the entire control group).

Microarray analysis of KEB-7 and EBDM-1 cells showed increased expression of MMPs 1, 7, 9, 13 and 19 compared to NEB-1 wild-type keratinocytes (Table 3). To verify these data, we performed SQRT-PCR and found each of the above MMPs to be significantly increased at the mRNA level, except *MMP-1* in the KEB-7 cell line and *MMP-13* in the EBDM-1 cell line. Moreover, *MMP-7* (matrilysin) and *MMP-13* (collagenase-3) were expressed at the highest levels in KEB-7 cells, and, *MMP-9* (gelatinase B) and *MMP-19* were expressed at the highest levels in EBDM-1 cells (Fig. 2A). We analyzed MMP-7 and MMP-9 protein expression in 48-h-conditioned cell culture supernatant by ELISA. We only found MMP-9 levels to be increased in both patient cell lines, by more than 2-fold in KEB-7 and by 46-fold in EBDM-1 cells compared to NEB-1 (Fig. 2B). MMP-7 was not increased significantly on the protein level in KEB-7 or EBDM-1 (data not shown). We further analysed MMP-9 levels *in vivo* and found significantly increased levels in EBS patients blister fluids vs. healthy controls (Fig. 2C).

**Table 3:** Microarray analysis of matrix metalloproteinases.

		KEB7 vs. NEB1		EBDM1 vs. NEB1	
Ref Seq	Gene	Sig log ratio	Fold change	Sig log ratio	Fold change
NM_002421	MMP1	0.98	1.97	1.87	3.66
NM_004530	MMP2	−0.22	−1.17	−0.83	−1.77
NM_002422	MMP3	−0.04	−1.03	0.08	1.06
NM_002423	MMP7	1.58	3.00	−2.99	−7.95
NM_002424	MMP8	0.08	1.06	0.09	1.06
NM_004994	MMP9	1.67	3.18	1.21	2.31
NM_002425	MMP10	−0.04	−1.03	0.43	1.35
NM_005940	MMP11	0.00	1.00	0.09	1.07
NM_002426	MMP12	0.08	1.06	−0.67	−1.59
NM_002427	MMP13	0.98	1.97	−0.05	−1.04
NM_004995	MMP14 (MT1-MMP)	−0.13	−1.10	−0.03	−1.02
NM_002428	MMP15 (MT2-MMP)	−0.34	−1.26	−0.37	−1.29
NM_005941	MMP16 (MT3-MMP)	0.09	1.07	−0.12	−1.08
NM_016155	MMP17 (MT4-MMP)	0.06	1.04	−0.05	−1.03
NM_002429	MMP19	1.75	3.35	0.92	1.89
NM_004771	MMP20	−0.06	−1.05	0.00	−1.00
NM_147191	MMP21	0.10	1.07	0.05	1.04
NM_006983	MMP23B	0.51	1.42	0.06	1.04
NM_006690	MMP24 (MT5-MMP)	−0.12	−1.09	−0.08	−1.06
NM_022468	MMP25 (MT6-MMP)	0.48	1.39	0.22	1.16
NM_021801	MMP26	−0.17	−1.12	0.16	1.12
NM_022122	MMP27 (MMP22)	−0.06	−1.04	0.04	1.03
NM_024302	MMP28	0.66	1.58	−0.94	−1.91

### Expression of the Cdc42 Guanine Nucleotide Exchange Factor *ARHGEF9* was Upregulated in EBS-DM Cell Lines and it Activated Cdc42

**Figure 3:**
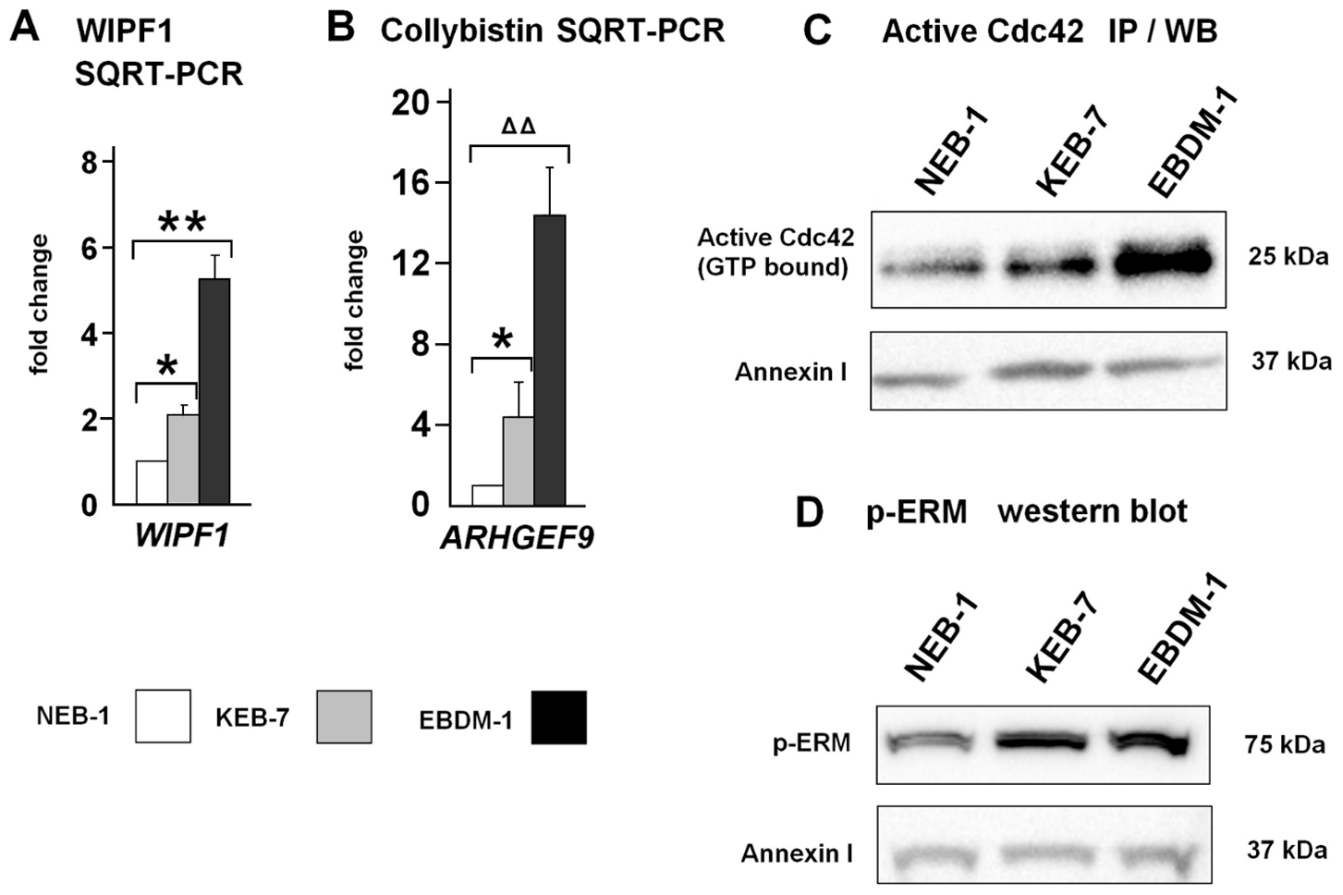
Aberrant regulation of actin cytoskeleton components. **A.** SQRT-PCR shows increased *WIPF1* mRNA expression in KEB-7 and EBDM-1 cell lines compared to NEB-1 wild-type keratinocytes (n = 3). **B.** SQRT-PCR shows increased *ARHGEF9* mRNA expression in KEB-7 and EBDM-1 cell lines compared to NEB-1 wild-type keratinocytes (n = 5). **C.** Immunoprecipitation and western blot show increased amounts of active (GTP-bound) Cdc42 protein in KEB-7 and EBDM-1. Annexin-I was used as a loading control. **D.** Western blot of whole-cell lysates shows increased amounts of phospho-ERM proteins in KEB-7 and EBDM-1. Annexin-I was used as a loading control. Student’s *t*-test was performed with p values: * ≤0.05, ** ≤0.01, ΔΔ ≤0.0005.

As outlined above, Wally et al. observed increased invasiveness of KEB-7 and EBDM-1 cells compared to NEB-1 in Matrigel™ invasion chambers [12]. When considering cell migration and invasiveness, the polymerization of actin filaments has to be taken into account. Microarray analysis showed no increased expression of the actin gene itself or of most factors that contribute to actin polymerization like RhoA, Rac1 or Cdc42. However, in KEB-7 cells, we found clear upregulation of the *WIPF1* gene (Table 4; Fig. 3A), which binds to the Wiskott-Aldrich Syndrome protein (WASP) and plays an important role in actin cytoskeleton dynamics.

**Table 4:** Microarray analysis of genes involved in actin cytoskeleton dynamics.

		KEB7 vs. NEB1		EBDM1 vs. NEB1	
Ref Seq	Gene	Sig log ratio	Fold change	Sig log ratio	Fold change
NM_001100	ACTA1	−0.45	−1.36	−0.05	−1.04
NM_001141945	ACTA2	0.25	1.19	0.03	1.02
NM_001101	ACTB	−0.07	−1.05	−0.01	−1.01
NM_001017992	ACTBL2	0.63	1.55	−0.73	−1.66
NM_005159	ACTC1	−0.22	−1.16	−0.25	−1.19
NM_001005386	ACTR2 (Arp2)	−0.06	−1.04	0.09	1.06
NM_005721	ACTR3 (Arp3)	−0.49	−1.40	0.18	1.14
NM_032995	ARHGEF4	2.01	4.03	0.18	1.14
NM_015185	ARHGEF9	3.24	9.44	1.90	3.74
NM_001001669	ARHGEF37	2.04	4.12	−0.67	−1.59
NM_044472	CDC42	0.27	1.21	0.27	1.21
NM_003607	CDC42BPA	0.75	1.68	0.73	1.66
NM_006035	CDC42BPB	0.60	1.51	0.09	1.07
NM_017525	CDC42BPG	0.98	1.98	−0.07	−1.05
NM_003379	EZR (ezrin)	−0.86	−1.81	0.16	1.12
NM_002444	MSN (moesin)	0.06	1.04	0.66	1.59
NM_018890	RAC1	−0.05	−1.03	0.15	1.11
NM_002906	RDX (radixin)	0.34	1.27	0.18	1.14
NM_001664	RHOA	−0.18	−1.14	0.25	1.19
NM_003931	WASF1	0.62	1.54	0.35	1.27
NM_006990	WASF2	0.13	1.10	0.15	1.11
NM_006646	WASF3	0.83	1.77	0.12	1.09
NM_000377	WAS	−0.12	−1.09	0.18	1.13
NM_003387	WIPF1	1.29	2.45	0.22	1.16

Our attention was drawn to the upregulation of two Rho guanine nucleotide exchange factors (GEFs) (*ARHGEF4*, *ARHGEF37*) and one Cdc42 GEF (*ARHGEF9*) (Table 4). We chose *ARHGEF9* for further analysis with SQRT-PCR because it was upregulated in both cell lines and its mRNA levels were increased more than 4-fold in KEB-7 and more than 14-fold in EBDM-1 (Fig. 3B). ARHGEF9 (collybistin) is the guanine nucleotide exchange factor responsible for GDP-GTP exchange of the small GTPase Cdc42 and is mainly found in the brain, heart and skeletal muscle [15]. The observed upregulation of the *ARHGEF9* gene adds to our understanding of the increased invasiveness of the two EBS-DM cell lines, presumably due to downstream activation of the Cdc42 signaling cascade.

Like other small GTPases, Cdc42 can be present in an active (GTP-bound) or inactive (GDP-bound) state [16]. To investigate the levels of active Cdc42 we performed immunoprecipitations of lysates of NEB-1, KEB-7 and EBDM-1 cells using an antibody that binds only the activated GTP-bound form of Cdc42. The precipitates were then subjected to SDS-PAGE and western blot analysis using an antibody that detects both active and inactive forms of Cdc42. We observed higher amounts of activated Cdc42 protein in KEB-7 and EBDM-1 cells compared to wild-type NEB-1 cells (Fig. 3C). Therefore, it has to be considered that elevated levels of activated Cdc42 protein could influence Cdc42 downstream targets and thereby alter actin cytoskeleton dynamics.

### Phosphorylation of ezrin/radixin/moesin (ERM) was Increased in EBS-DM Cell Lines

One of the downstream targets of Cdc42 is the myotonic dystrophy kinase-related Cdc42-binding kinase (MRCKα,-β and -γ) [17]. When MRCKs are activated by Cdc42 they phosphorylate and thereby activate downstream targets like the ERM proteins (ezrin/radixin/moesin) [18].

The genes coding for MRCKs (*CDC42BPA*, *CDC42BPB*, *CDC42BPG*) and the genes coding for ERM proteins were not significantly upregulated above a two-fold threshold in the microarray analysis (Table 4). Only *CDC42BPG* showed a considerable upregulation of 1.98-fold in KEB-7 compared to NEB-1. However, downstream activation of the Cdc42 pathway can be analyzed by determining the phosphorylation status of ERM proteins. Using an antibody against phosphorylated ERM proteins (75–80 kDa), we were able to show that phospho-ERM proteins are indeed present in significantly higher amounts in KEB-7 and EBDM-1 cells compared to NEB-1 cells (Fig. 3D). Determination of the protein amounts using *BIO-RAD* Image Lab 3.0.1 software revealed a 30–40% increase in phospho-ERM protein levels in the EBS-DM cell lines.

### KEB-7 and EBDM-1 Showed Deregulation of Cytokeratin Expression

**Figure 4:**
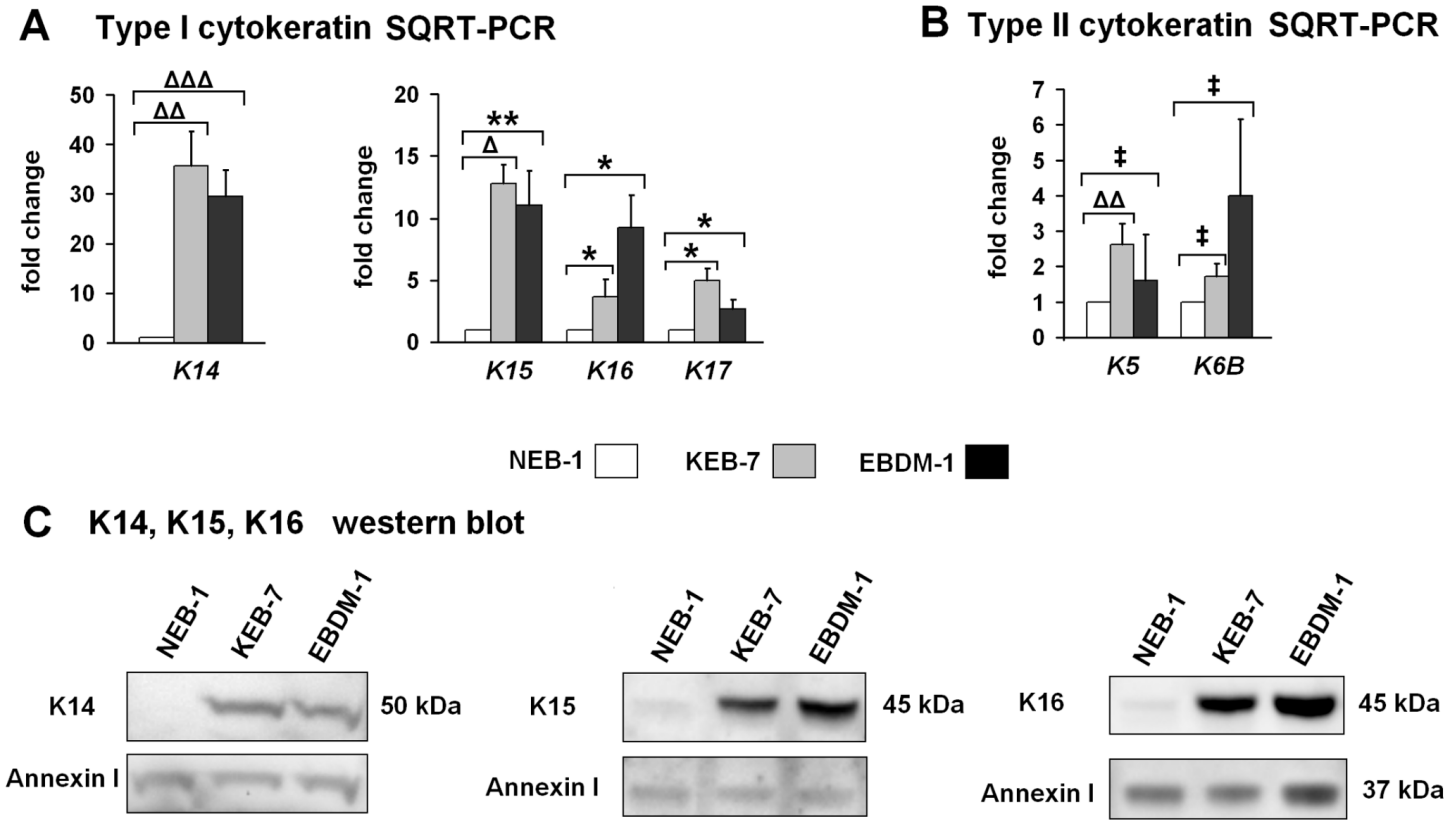
Type I and type II cytokeratin expression. **A.** SQRT-PCR shows an increase in mRNA expression of type I cytokeratins *K14*, *15*, *16* and *17* in KEB-7 (K14 R125P) and EBDM-1 (K14 R125H) cell lines compared to NEB-1 wild-type keratinocytes (n = 4 to n = 7). **B.** SQRT-PCR of type II cytokeratins shows only a significant increase of *K5* mRNA expression in the KEB-7 cell line (n = 3 to n = 5). **C.** Western blot analysis from whole-cell lysates revealed increased protein expression of K14, 15 and 16 in both EBS-DM cell lines. Annexin-I was used as a loading control. Student’s *t*-test was performed with p values: * ≤0.05, ** ≤0.01, Δ≤0.001, ΔΔ≤0.0005, ΔΔΔ≤0.0001, ‡ = no significant difference between investigated cell lines.

The microarray data indicated type I and type II cytokeratins to be differentially regulated in the two investigated K14 mutant cell lines (Table 5), which confirms previous investigations [12], [19]. Using SQRT-PCR, we measured the expression of cytokeratins that are related to EBS (K5, K14) as well as cytokeratins that are expressed in activated keratinocytes (K6, K16, K17) or under specific cellular conditions (K15). The mRNA levels of *K14*, *K15*, *K16* and *K17* were significantly increased in KEB-7 and EBDM-1 cells (Fig. 4A). Although increased mRNA levels of *K5* and *K6B* were seen in the microarray analysis, only *K5* was verified to be significantly upregulated in KEB-7 (Fig. 4B). We investigated the protein levels of K14, K15 and K16, and all three cytokeratins were increased significantly in western blot analysis in both Dowling-Meara cell lines (Fig. 4C).

**Table 5:** Microarray analysis of cytokeratin genes.

		KEB7 vs. NEB1		EBDM1 vs. NEB1	
Ref Seq	Gene	Sig logratio	Foldchange	Sig logratio	Foldchange
NM_006121	KRT1	3.25	9.52	0.31	1.24
NM_000424	KRT5	0.37	1.30	0.17	1.12
NM_005554	KRT6A	0.23	1.17	0.57	1.49
NM_005555	KRT6B	1.76	3.40	0.83	1.78
NM_002273	KRT8	−1.49	−2.81	−0.54	−1.45
NM_000421	KRT10	0.18	1.13	−2.74	−6.67
NM_000526	KRT14	3.59	12.0	2.15	4.44
NM_002275	KRT15	3.71	13.1	0.35	1.27
NM_005557	KRT16	3.58	11.9	1.04	2.06
NM_000422	KRT17	1.80	3.49	0.59	1.51
NM_199187	KRT18	−1.30	−2.46	0.09	1.07

### Expression of Junction Proteins was Increased in EBS-DM Cell Lines

**Figure 5:**
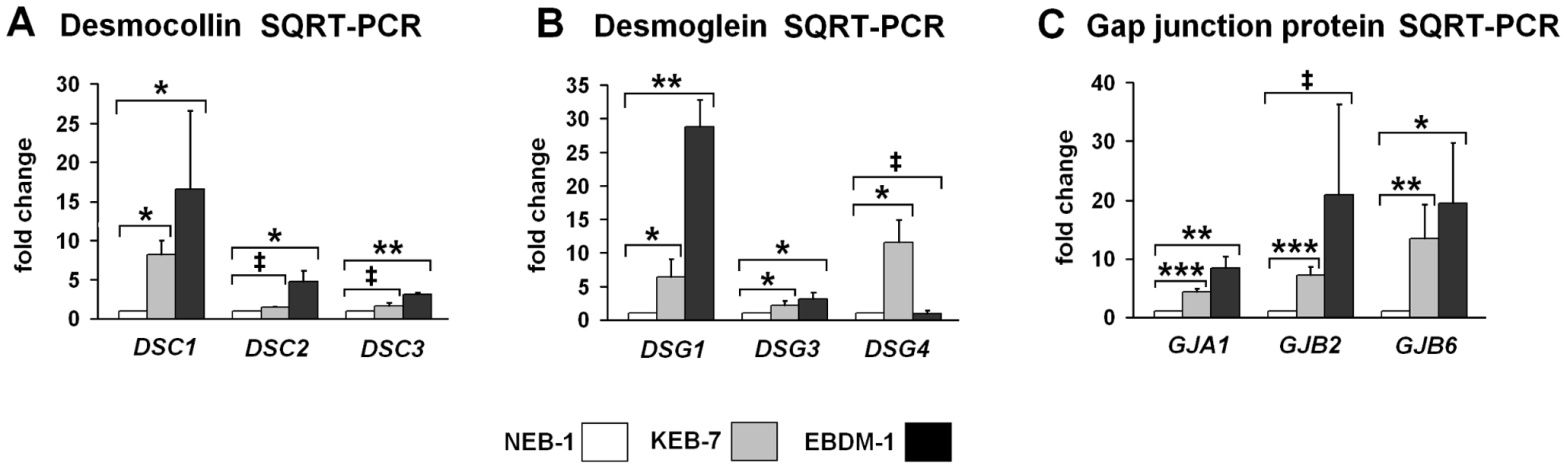
Expression of junction proteins in EBS-DM cell lines. **A.** SQRT-PCR of desmocollin mRNA expression in NEB-1, KEB-7 and EBDM-1 (n = 3 to n = 4). *DSC1* shows the highest increase in both EBS-DM cell lines. *DSC2* and *DSC3* are increased only in EBDM-1 but not in KEB-7. **B.** SQRT-PCR of desmoglein mRNA expression in NEB-1, KEB-7 and EBDM-1 (n = 3 to n = 5). *DSG1* shows the highest increase in EBDM-1. Only a slight increase was observed for *DSG3*. *DSG4* is only significantly increased in KEB-7. **C.** SQRT-PCR of gap junction protein mRNA expression in NEB-1, KEB-7 and EBDM-1 (n = 3 to n = 5). *GJA1* and *GJB6* expression is increased in both EBS-DM cell lines. *GJB2* is only increased significantly in KEB-7. Student’s *t*-test was performed with p values: * ≤0.05, ** ≤0.01, *** ≤0.005, ‡ = no significant difference between investigated cell lines.

Bioinformatic analysis of the microarray data revealed junction proteins as one major group of regulated genes (Table 6). We verified these data with SQRT-PCR for the desmocollins *DSC1*, *DSC2* and *DSC3* (Fig. 5A), the desmogleins *DSG1*, *DSG3* and *DSG4* (Fig. 5B), and the gap junction proteins *GJA1*, *GJB2* and *GJB6* (Fig. 5C) because they all showed significant upregulation in the array. The highest transcript levels were found for *DSC1*, *DSG1* and *GJB6* (connexin 30), with an increased expression of 15- to almost 30-fold compared to NEB-1 wild-type keratinocytes.

**Table 6:** Microarray analysis of junction protein genes.

		KEB7 vs. NEB1		EBDM1 vs. NEB1	
Ref Seq	Gene	Sig logratio	Foldchange	Sig logratio	Foldchange
NM_024421	DSC1	1.87	3.65	0.47	1.39
NM_024422	DSC2	1.13	2.18	1.02	2.03
NM_024423	DSC3	1.49	2.81	0.68	1.60
NM_001942	DSG1	3.50	11.3	1.05	2.07
NM_001943	DSG2	0.07	1.05	0.26	1.20
NM_001944	DSG3	1.17	2.25	0.74	1.67
NM_001134453	DSG4	2.09	4.26	0.25	1.19
NM_000165	GJA1	1.53	2.88	1.68	3.21
NM_021954	GJA3	0.22	1.16	0.03	1.02
NM_002060	GJA4	−0.07	−1.05	−0.08	−1.06
NM_005266	GJA5	0.60	1.52	0.16	1.12
NM_005267	GJA8	−0.04	−1.03	0.01	1.01
NM_030772	GJA9	−0.13	−1.10	0.17	1.13
NM_032602	GJA10	−0.14	−1.10	0.15	1.11
NM_000166	GJB1	0.24	1.18	−0.08	−1.06
NM_004004	GJB2	2.15	4.43	1.10	2.15
NM_024009	GJB3	0.79	1.73	0.56	1.47
NM_153212	GJB4	1.03	2.04	−0.12	−1.09
NM_005268	GJB5	0.72	1.65	0.43	1.34
NM_001110219	GJB6	3.41	10.6	1.50	2.82
NM_198568	GJB7	−0.08	−1.05	0.22	1.17

### CXCL8/IL-8 Expression was Increased in KEB-7, and High Levels were Found in EBS Patients Blister Fluids

**Figure 6:**
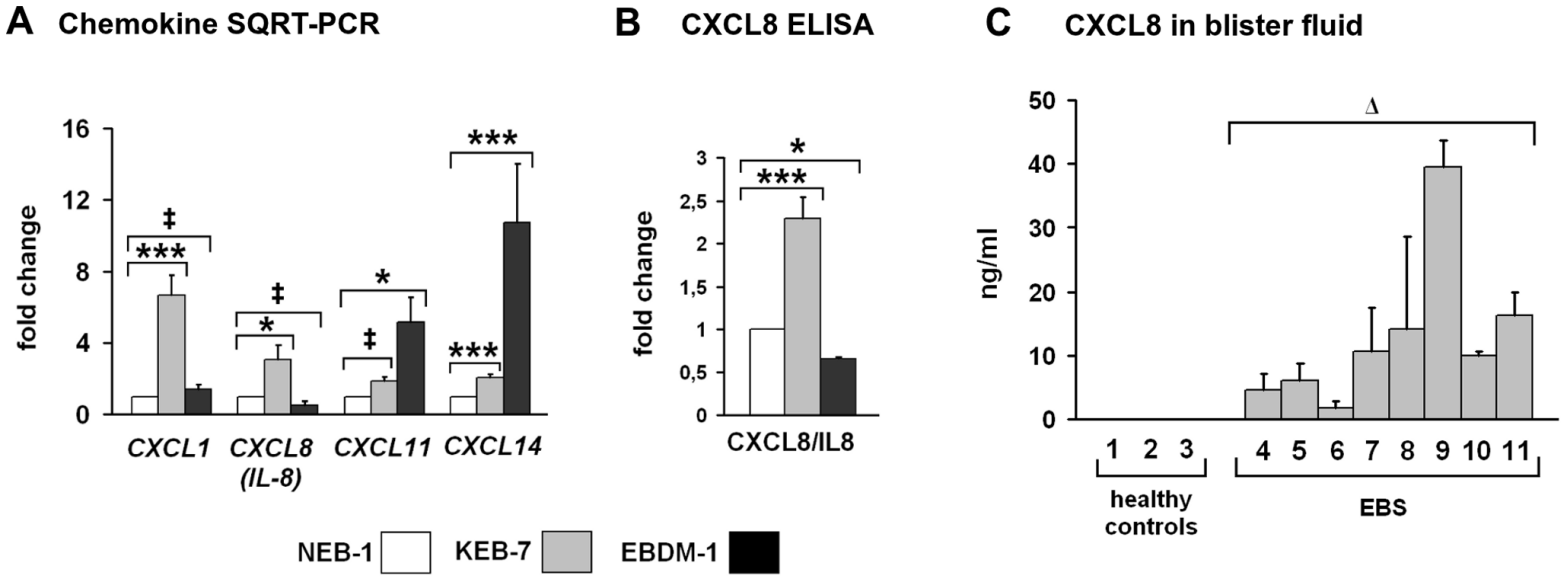
Expression of chemokines. **A.** SQRT-PCR of chemokine mRNA expression in NEB-1, KEB-7 and EBDM-1 (n = 3 to n = 5). *CXCL1*, *CXCL8/IL-8* and *CXCL14* expression was increased in KEB-7. Only *CXCL11* and *CXCL14* were increased in EBDM-1. **B.** CXCL8/IL-8 ELISA of 48-h-conditioned cell culture supernatant showed 2-fold upregulation at the protein level in KEB-7 but not in EBDM-1, which correlates with the SQRT-PCR results (n = 4). **C.** CXCL8/IL-8 concentrations were highly increased in EBS patients blister fluids. In blister fluids of healthy controls no CXCL8/IL-8 was detectable (n = 3 to n = 4). The numbers correlate with Table 1. Student’s *t*-test was performed with p values: * ≤0.05, *** ≤0.005, Δ≤0.001, ‡ = no significant difference between investigated cell lines. (In 6C, the Student’s *t*-test compared the entire patient group to the entire control group).

Blister formation is often accompanied by infiltration of cells of the immune system into the skin. These immune cells are recruited by chemokines. Highly increased expression of the chemokines *CXCL1*, *CXCL8*/*IL-8* and *CXCL14* was evident in KEB-7 cells and of *CXCL11* in EBDM-1 cells (Table 7). We used SQRT-PCR to confirm the upregulation of *CXCL1*, *CXCL8/IL-8* and *CXCL14* in the KEB-7 cell line. *CXCL11* was not upregulated above the 2-fold threshold in KEB-7. *CXCL11* and *CXCL14* were increased significantly in EBDM-1, as determined by SQRT-PCR (Fig. 6A), although *CXCL14* was not increased in the microarray analysis. We determined the protein expression of all four chemokines in 48-h-conditioned cell culture supernatant with ELISA and found CXCL8/IL-8 levels to be increased by more than 2-fold in KEB-7 (Fig. 6B), thereby almost exactly correlating with the mRNA expression found in SQRT-PCR. We found no increased protein levels of CXCL1, CXCL11 or CXCL14 (data not shown). We further analysed blister fluids of EBS patients and found significantly increased levels of CXCL8/IL-8 compared to healthy controls (Fig. 6C).

**Table 7:** Microarray analysis of C-X-C chemokines.

		KEB7 vs. NEB1		EBDM1 vs. NEB1	
Ref Seq	Gene	Sig log ratio	Fold change	Sig log ratio	Fold change
NM_001511	CXCL1	1.78	3.43	0.22	1.17
NM_002089	CXCL2	0.68	1.61	0.51	1.42
NM_002090	CXCL3	0.16	1.11	0.23	1.18
NM_002619	CXCL4 (PF4)	0.23	1.17	−0.02	−1.02
NM_002994	CXCL5	0.13	1.09	−0.30	−1.23
NM_002993	CXCL6	−0.46	−1.37	−0.13	−1.09
NM_002704	CXCL7 (PPBP)	−0.05	−1.04	0.29	1.22
NM_000584	CXCL8 (IL8)	1.98	3.94	−1.21	−2.31
NM_002416	CXCL9	−0.27	−1.21	0.02	1.01
NM_001565	CXCL10	0.40	1.32	0.33	1.26
NM_005409	CXCL11	0.10	1.07	1.43	2.69
NM_000609	CXCL12	−0.33	−1.25	−0.07	−1.05
NM_006419	CXCL13	−0.04	−1.03	−0.09	−1.06
NM_004887	CXCL14	5.15	35.4	−0.64	−1.55
NM_022059	CXCL16	0.60	1.51	0.04	1.03
NM_198477	CXCL17	0.09	1.07	−0.33	−1.25

In the microarray analysis, neither C-chemokines (XCL1, XCL2), C-C chemokines (CCL1, -28) nor CX3CL1 showed any up- or downregulation and none of the corresponding receptors was differentially regulated in KEB-7 or EBDM-1 (data not shown).

### Incubation of EBDM-1 Cells with IL-1β Neutralizing Antibody Reduced the Expression of Target Genes

**Figure 7:**
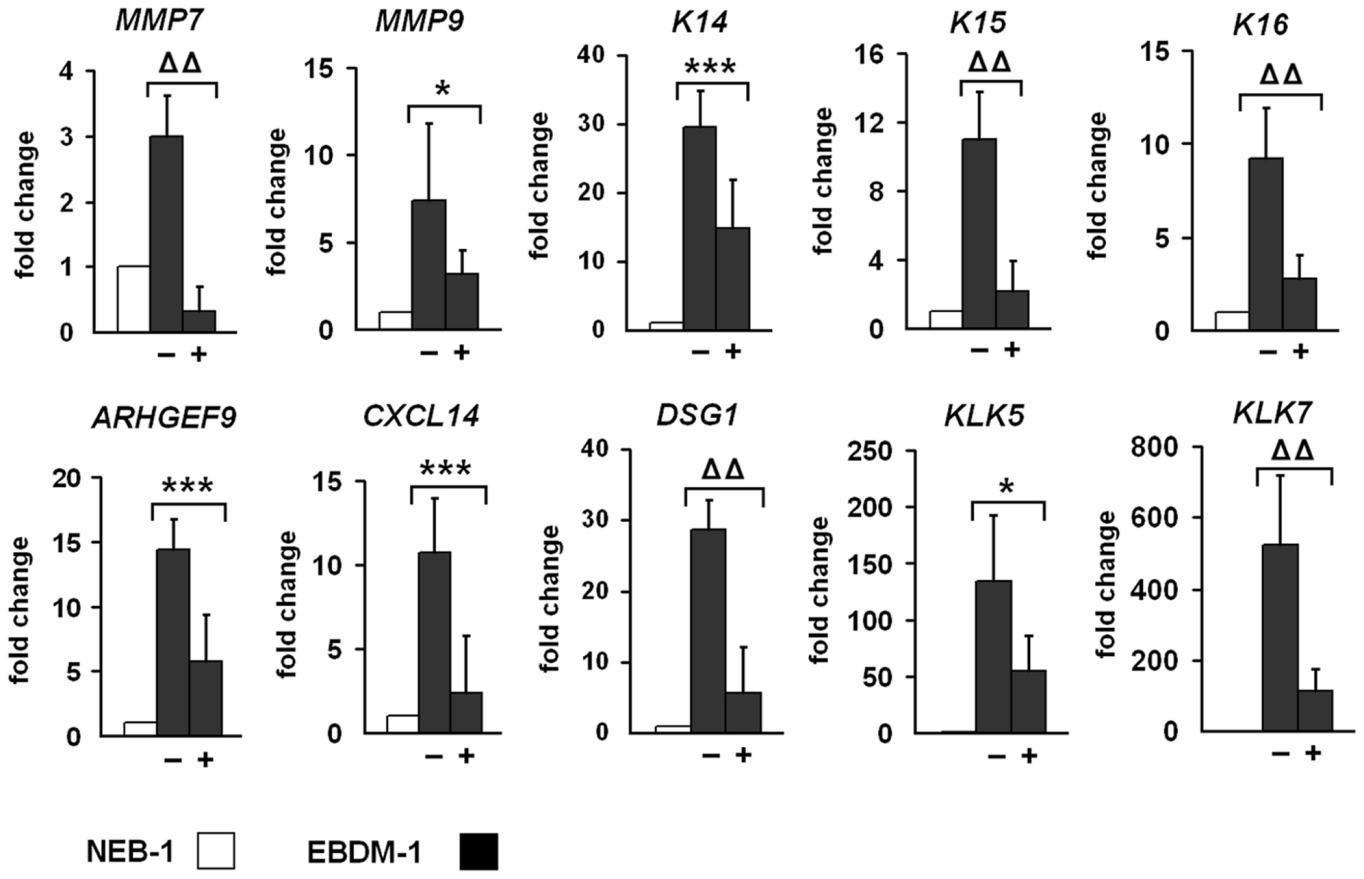
Reduced expression of target genes upon IL-1β depletion. NEB-1 cells and EBDM-1 cells were incubated with 2 µg/ml IL-1β neutralizing antibody for 24 h to deplete IL-1β in the culture medium. Expression of untreated EBDM-1 cells **(−)** was compared to treated EBDM-1 cells **(+)** by SQRT-PCR. We picked at least one representative target gene of each of the six functional groups identified in the microarray. All investigated targets showed significant downregulation after IL-1β depletion. No differences in geneexpression were observed in NEB-1 cells −/+ antibody incubation (n = 3). Student’s *t*-test was performed with p values: * ≤0.05, *** ≤0.005, ΔΔ ≤0.0005.

To study the role of IL-1β and its ability to alter the gene expression profile, we incubated EBDM-1 cells with IL-1β neutralizing antibody (2 µg/ml, R&D Systems) for 24 h and analyzed mRNA expression of distinct target genes by using SQRT-PCR. We chose EBDM-1 because it showed the most severe phenotype in most of our experiments. As targets we chose at least one representative of each of the six groups of regulated genes identified in our microarray analysis. In three independent experiments and with at least four SQRT-PCR runs per experiment, we observed a significant reduction of gene expression after IL-1β antibody incubation for all of the investigated target genes (Fig. 7).

## Discussion

### The Potential Role of MMP-9 in Blister Formation

Reduction of blister formation was reported in a small group of EBS patients after administration of tetracycline orally over a period of several weeks [20], [21]. The patients comprised a heterogeneous group that included Dowling-Meara and EBS-localized patients (formerly known as Weber-Cockayne). The molecular mechanism by which tetracycline may reduce blister formation is still speculative, but the drug and especially its derivative doxycycline are known inhibitors of MMPs [22]. This indicates a potential causal link between blister formation due to increased expression of MMPs and its amelioration by tetracycline treatment.

Although a potential role of MMP-9 in blister formation is currently discussed for Pemphigus vulgaris [23], a correlation with blister formation in EBS has never been considered so far. The *in vitro* and *in vivo* data of increased MMP-9 expression, that we present here, may contribute to a better understanding of the EBS pathophysiology, which hopefully leads to new therapeutic options.

### CXCL8/IL-8 Expression and Blister Formation

A potential role of CXCL8/IL-8 in blister formation has already been established. In the autoimmune blistering disease bullous pemphigoid (BP), infiltration of neutrophils into the skin is required for blister formation. The recruitment of neutrophil granulocytes is stimulated by IL-8 as a consequence of the binding of autoantibodies to the hemidesmosomal 180-kD BP autoantigen (BP180). In a mouse model, inhibition of neutrophil infiltration to the skin as well as inhibition of IL-8 circumvented blister formation [24].

In the present study, we observed significantly increased levels of CXCL8/IL-8 in EBS patients blister fluids. Based on these new data, CXCL8/IL-8 should be considered as a new and potential therapeutic target of blistering diseases. In *in vitro* studies it was shown that IL-8 release from cultured human keratinocytes is inhibited by the antibiotic molecule dapsone [25]. Inhibition of IL-8 and stopping of immune cell infiltration using small molecules, either already available like dapsone or yet to be developed, should open a therapeutic window into the treatment of blistering diseases like EBS-DM.

### Kallikrein Expression in EBS-DM Cell Lines

The increased expression of KLK5 and KLK7 in the two EBS-DM cell lines KEB-7 and EBDM-1 indicated a potential role in the pathophysiology of Dowling-Meara-related blister formation. Hypothetically, kallikreins could play a role in acantholysis, the separation of cell-cell contacts, but there are only few reports of Dowling-Meara patients that show signs of acantholysis [26], [27]. Nevertheless, since we did not observe any differences in KLK5 and KLK7 expression in patients blister fluids vs. controls, their role as potential therapeutic targets can not be suggested.

As the expression of kallikreins as well as of junction proteins could be a sign of keratinocyte terminal differentiation, we analysed the microarray data for differentiation-related genes, such as involucrin (IVL), filaggrin (FLG), loricrin (LOR), small proline-rich proteins (SPRR) and SPINK5/LEKTI (data not shown). Out of 14 investigated genes, 6 were upregulated in KEB-7 but none were upregulated in EBDM-1. We analysed these genes with SQRT-PCR and found IVL and FLG increased in KEB-7 and SPINK5/LEKTI in EBDM-1. Western blot confirmed the increase of IVL in KEB-7. FLG could not be detected on the protein level, and SPINK5/LEKTI showed no differences between patient cell lines vs. NEB-1, or between EBS patients blister fluids vs. controls. The fact, that differentiation-related proteins were expressed in only one cell line, but most of the targets were expressed in both cell lines, does not support the idea of a pure cell culture artefact due to keratinocyte differentiation.

### The Role of Cytokeratins and Junction Proteins in Blistering Diseases

In basal cells of epithelia, K5 and K14 are the characteristic keratins expressed throughout the lifetime of the cell in the normal healthy state. The observed upregulation of K15 in EBS-DM cell lines occurs due to insufficient K14 function; however, K15 is unable to fully compensate the lack of K14 both in the recessive forms of EBS and in the dominant negative forms such as Dowling-Meara [28]. A change in keratin expression also appears after injury of the skin. In this case, the keratinocyte activation cycle is launched and K6, K16 and K17 are expressed, and the cells exhibit increased migratory potential necessary for wound closure [7]. The upregulation of K16 and K17 in EBS-DM cell lines, as shown in the present study as well as in another recent study [19], indicates that these cell lines are in an activated state as if undergoing constant wounding. It was also shown recently that the increased migratory potential of KEB-7 cells can be reduced significantly by genetic correction of the K14 R125P mutation at the mRNA level by using SMaRT technology [29]. This indicates a potential role of K14 mutations in the mechanisms leading to the migration- and invasion phenotypes of keratinocytes *in vitro*.

It was shown in several studies that IF stability is dependent on desmosome integrity, and that perturbations of desmosomes lead to a retraction of the IF network toward the nucleus [30], [31]. Such accumulations of keratin around the nucleus were also found in Dowling-Meara cell lines [32]. Furthermore, downregulation of certain junction proteins such as desmoplakin and plakoglobin was observed in cell lines carrying severe K5 and K14 mutations [33]. Based on these data it can be hypothesised, that in keratin-associated blistering diseases a dysregulation of keratin expression correlates with a dysregulation of junction protein expression.

### The Validity of *in vivo* Data

In the present study, we compared the blister fluids of EBS patients with the blister fluids of healthy controls. Considering healthy controls the aetiology and the mechanism of blister formation can be different from EBS patients and has to be taken into account for interpretation of results. E. g. the skin-layer in which blister formation occurs can be different from that in EBS patients and blisters can arise with or without signs of inflammation. For control number 1, blister fluid was obtained from a bed-ridden elderly male person that developed a mechanical (tension-) blister in the inguinal region in the course of mobilization procedures during patient care. The person is considered healthy in the sense of non-EB and without any bullous or other skin diseases. For control number 2, mechanical (friction-) blisters were induced deliberately on both heels by a healthy volunteer (non-EB, no skin diseases) from our lab by wearing unfitting shoes. Finally, considering control number 3, a blister developed on the foot of a healthy person (non-EB, no skin diseases) after a burn accident. No traces of blood and no signs of inflammation, pruritus or unusual pain occurred with the blisters of the three healthy controls. Taken together, by comparison of EBS blister fluids with the control samples described above, we consider the obtained *in vivo* data to be valid.

### Molecular Pathomechanisms in Dowling-Meara: IL-1β, MMP-9 and CXCL8/IL-8

In the study of Wally et al., IL-1β was shown to be the inaugurating mediator of the Dowling-Meara phenotype observed in the EBS-DM cell lines KEB-7 and EBDM-1. The same study showed activation of the JNK stress pathway through IL-1β, and amelioration of the phenotype *in vitro* by depleting IL-1β with a neutralizing antibody [12]. Based on that knowledge, the present study investigated the gene and protein expression profiles of KEB-7 and EBDM-1 and showed that expression of these genes is dependent on IL-1β signaling.

**Figure 8:**
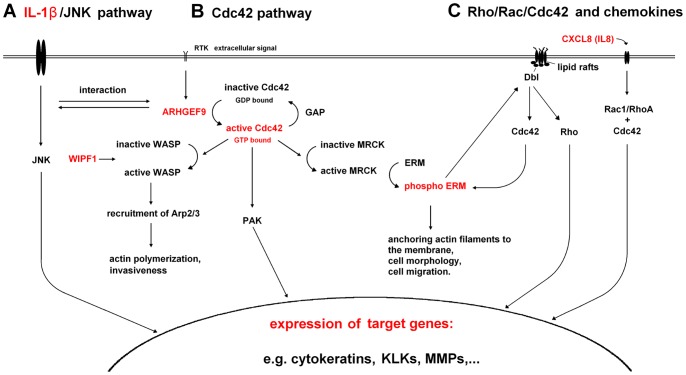
Schematic representation of pathways involved in EBS-DM pathomechanisms. **A.** IL-1β activates the JNK pathway in Dowling-Meara cell lines and induces expression of target genes. The IL-1 pathway also interacts with the Cdc42 pathway. **B.** Schematic representation of the Cdc42 pathway. Upregulation of collybistin (ARHGEF9) leads to increased GDP-GTP exchange and activation of Cdc42. Active Cdc42 activates its downstream effectors like WASP (Wiskott-Aldrich Syndrome Protein) and Arp2/3 (Actin-related protein 2 and 3) as well as MRCK and ERM proteins [41], [42], [43]. The Arp2/3 complex serves as a nucleation core for actin polymerization, and phospho-ERM proteins connect actin filaments to the plasma membrane. *WIPF1* expression was also upregulated in EBS-DM cell lines. The Cdc42 downstream-effects contribute to actin dynamics, invasiveness and, through PAK, to the gene expression levels. **C.** Depiction of the interconnected network of small GTPases like Rho, Rac and Cdc42 as well as CXCL8/IL-8. Activated Cdc42 leads to increased amounts of phospho-ezrin. After activation, p-ezrin recruits the GEF Dbl to lipid rafts and induces activation of both Cdc42 in a feedback loop as well as the Rho pathway. Binding of CXCL8/IL-8 also leads to activation of Rac1/RhoA and Cdc42, also resulting in expression of target genes. (Red colored items indicate aspects investigated in the present study).

Of course, the question remains, how does IL-1β mediate these effects and how are the molecular mechanisms interconnected? Fig. 8 shows an overview of the pathways described below. In the case of the Cdc42 pathway, inflammatory cytokines such as TNFα and IL-1 were shown to influence actin cytoskeleton dynamics by activation of Cdc42, and interconnections between IL-1 and Cdc42 signaling are known [34], [35] (Fig. 8A). Cdc42 activates downstream targets like the ERM proteins (Fig. 8B), and phospho-ezrin recruits the GEF Dbl to lipid rafts and induces the activation of Cdc42 in a positive feedback loop [36] (Fig. 8C). Cdc42 also activates the Rac and Rho pathways as well as PAK (p21-activated kinase), and activates downstream effects that lead to changes in gene expression [35]. Besides their interconnections, the IL-1 and Rho pathways can also act independently from each other [37]. Rho pathways like Rac1, RhoA and Cdc42 are also activated by IL-8 [38] (Fig. 8C), which we showed to be upregulated in EBS-DM cell lines and in patients blister fluids.

Expression of gelatinases and activation of MMP-9 and MMP-3 was shown to be induced by IL-1α in a dose-dependent manner in chondrocytes [39]. IL-1β activates the JNK pathway. It was shown in an *in vitro* model, that JNK components and RhoGTPases interact through crosstalk and are necessary for induction of MMP-9 expression in wounded keratinocytes [40].

The evident deregulation of different pathways like IL-1β and Cdc42 in K14 mutant cell lines and our findings *in vivo* demand a new hypothesis that gives greater weight to the role of matrix metalloproteinases and chemokines in blister formation. The idea of interactions between IL-1β, MMP-9 and CXCL8/IL-8 is supported by the literature, and the findings presented in our study provide a better understanding of the disease mechanisms, which is a necessity for the development of new therapies.
